# Growth Differentiation Factor 15 as a Marker for Chronic Ventricular Pacing

**DOI:** 10.3390/jcm13247748

**Published:** 2024-12-18

**Authors:** Christoph Edlinger, Marwin Bannehr, Michael Lichtenauer, Vera Paar, Paulina Jankowska, Laurenz Hauptmann, Uta C. Hoppe, Christian Butter, Christiana Schernthaner

**Affiliations:** 1 Department of Cardiology, Heart Center Brandenburg, 16321 Bernau bei Berlin, Germanychristian.butter@immanuelalbertinen.de (C.B.); 2 Brandenburg Medical School (MHB) “Theodor Fontane”, 16816 Neuruppin, Germany; 3 Clinic of Internal Medicine II, Department of Cardiology, Paracelsus Medical University of Salzburg, 5020 Salzburg, Austriau.hoppe@salk.at (U.C.H.); c.schernthaner@salk.at (C.S.)

**Keywords:** GDF-15, pacemaker, heart failure

## Abstract

**Background/Objectives:** Right ventricular pacing is an effective and safe treatment option for patients experiencing symptomatic bradycardia. However, some individuals may develop left ventricular dysfunction as a consequence. Growth differentiation factor 15 (GDF-15), which is not present in a healthy adult heart, is upregulated in cardiomyocytes in response to various stress stimuli. This study aimed to explore the potential of GDF-15 as a biomarker for chronic right ventricular pacing. **Methods:** This single-center cross-sectional cohort study analyzed data from 265 consecutive patients (60.4% male) with either single- or dual-chamber pacemakers, all lacking pre-existing heart failure, who attended the outpatient department for routine follow-up. Chronic right ventricular (RV) pacing was defined as pacing exceeding 40% over the past year. Serum samples were collected, and GDF-15 levels were measured using a commercially available immunoassay (R&D Systems Inc., Minneapolis, MN, USA). Student’s *t*-test was utilized to assess group differences, and receiver operating characteristic (ROC) analysis was employed to evaluate diagnostic performance. **Results:** When stratifying patients by pacing burden, GDF-15 levels were significantly higher in those with pacing over 40% compared to those with 40% or less (789 ± 293 pg/mL vs. 1186 ± 592 pg/mL; *p* < 0.001). The ROC analysis indicated that GDF-15 serves as a marker for chronic RV pacing, yielding an area under the curve of 0.713 (95% confidence interval 0.650–0.776; *p* < 0.001). **Conclusions:** This study suggests that GDF-15 may be a valuable biomarker for chronic right ventricular pacing.

## 1. Introduction

Cardiac Pacemaker (PM) therapy is a very well-established standard procedure in cardiology that has been continuously improved over the years through both increasing clinical experience and technical development [1]. For the treatment of symptomatic bradycardia, apical placement of the right ventricular lead has been the preferred position for many years, which is a safe and comparatively easy-to-learn procedure. Nevertheless, this deep right apex positioning of the lead implicates a non-physiological stimulation site [2], which, especially in patients with a high ventricular pacing burden, might lead to a decrease in left ventricular ejection fraction (LVEF) [3,4,5,6,7,8]. According to previous literature, a decrease in LVEF might be expected with a right ventricular pacing (RVP) burden of >/=40. Recent studies have shown that even patients with a much lower RVP burden can potentially suffer significant deterioration [9,10]. The aim of modern pacemaker therapy is to influence the physiological stimulation of an individual patient “as little as possible but as much as necessary” [11,12]. Therefore, further developments such as cardiac resynchronization therapy (CRT) or, more recently, conduction pacing have been introduced. However, these treatment concepts require a certain amount of “know-how” and are associated with certain additional costs. Unfortunately, it is not possible to estimate with certainty in advance which patients will have a sustaining clinical benefit from an intensified treatment procedure such as CRT or conduction pacing. Moreover, in patients with already limited LVEF, during follow-up procedures, it is not always possible to certainly predict the impact of a high RVP on the etiology of heart failure [13]. For this reason, additional tools such as biomarkers could be helpful in order to be able to advise patients even more comprehensively in individual cases beyond clinical impressions. In recent years, a number of novel cardiac biomarkers have been developed that may be superior to classical cardiac biomarkers such as troponin or natriuretic peptides in estimating mortality, rehospitalization rates, and quality of life [14,15,16].

Growth differentiation factor-15 (GDF-15) is a cytokine member of the transforming growth factor β superfamily [17,18]. Under normal physiological conditions, it is only expressed in the placenta. However, it was also shown to be upregulated in response to various stimuli, including oxidative stress and cardiac pressure [19,20]. Additionally, circulating GDF-15 levels appear to positively correlate with the degree of myocardial fibrosis.

GDF-15 has a low biological fluctuation range, is stable during acute events, and has been investigated as a prognostic marker in patients with heart failure [21,22,23]. While RVP is an important and effective treatment in patients with atrioventricular block, it has been shown to promote left ventricular dysfunction and pacemaker-induced cardiomyopathy (PiCM) in up to 20% of patients with pacemakers [24]. Despite advances in and the benefits of treating arrhythmias with pacemakers, PiCM remains a public health problem with high morbidity and mortality since implant numbers are high and further increasing in an aging society [25]. Therefore, the early identification and risk stratification of high-risk patients with left ventricular (LV) dysfunction due to RVP are crucial.

Circulating biomarkers reflecting pathophysiological pathways involved in LV dysfunction development and progression may assist clinicians in the early diagnosis and management of those patients.

The aim of this study was to investigate the role of GDF-15 in comparatively “non-physiological” RVP. Our hypothesis was that GDF-15 might correlate with the RVP burden and serve as a potential tool for treatment decisions or patient monitoring.

## 2. Materials and Methods

In this single-center cross-sectional cohort study, data from 265 consecutive patients (60.4% male) with single- or dual-chamber pacemakers and no pre-existing heart failure who presented in the outpatient department for routine follow-up were analyzed.

The study has been approved by the local ethics committee (415-e/2101/5-2017, votum from 16 January 2017). All patients enrolled in the study were informed of the details prior to enrollment and had to sign a written informed consent form, which was approved in advance by the ethics committee. The study was conducted in accordance with the principles of the Declaration of Helsinki and Good Clinical Practice.

### 2.1. Study Population

**Figure 1:**
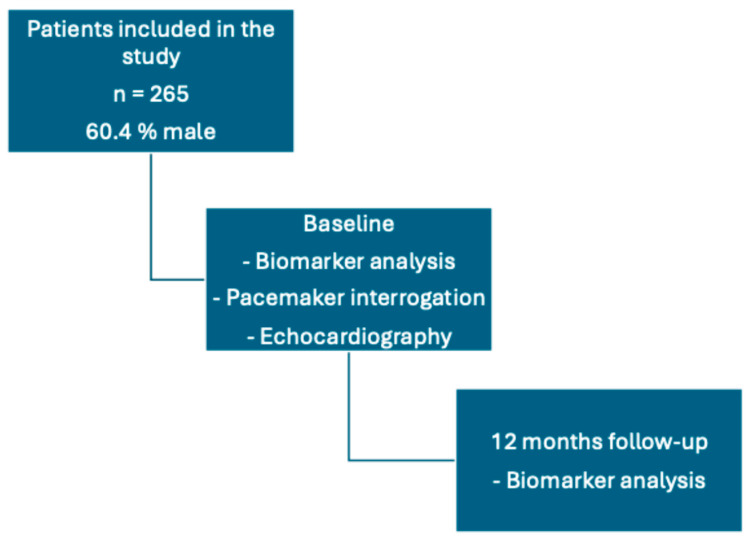
Patients’ flow through the study.

This is an allcomers study. Patients who attended our pacemaker outpatient clinic in a maximum care center aged over 18 years with a life expectancy of at least 1 year, and who were able to give consent were included. Patients who were not able to attend the follow-up (e.g., because of long travel distances) were excluded. All patients underwent routine pacemaker follow-up, and the RVP burden was recorded. In addition, transthoracic echocardiography (TTE) was performed to assess LVEF. Chronic RV pacing was defined as pacing burden > 40% within the last year. Figure 1 shows the patients’ flow through the study.

After local skin disinfection, a clean puncture of an arm vein was performed under usual venous stasis to obtain blood samples. After a follow-up of 12 months, another routine pacemaker follow-up was performed and blood samples were obtained.

### 2.2. Biomarker Analysis

Plasma samples were determined using a commercially available enzyme-linked immunosorbent assay (ELISA) kit (Human GDF-15 DuoSet ELISA, Catalog #: DY95 R&D Systems, Minneapolis, MN, USA). All experiments were performed according to the manufacturer’s instructions.

### 2.3. Statistical Analysis

All statistical analyses were performed using SPSS (Version 28.0, SPSS Inc., Armonk, NY, USA, 2021). The Shapiro–Wilk test was applied to test variables for normal distribution. Normally distributed metric data were expressed as mean ± standard deviation and analyzed using Student’s *t*-test.

Receiver operating characteristics (ROCs) were assessed to illustrate the diagnostic potential. A *p*-value < 0.05 was considered statistically significant.

## 3. Results

**Figure 2:**
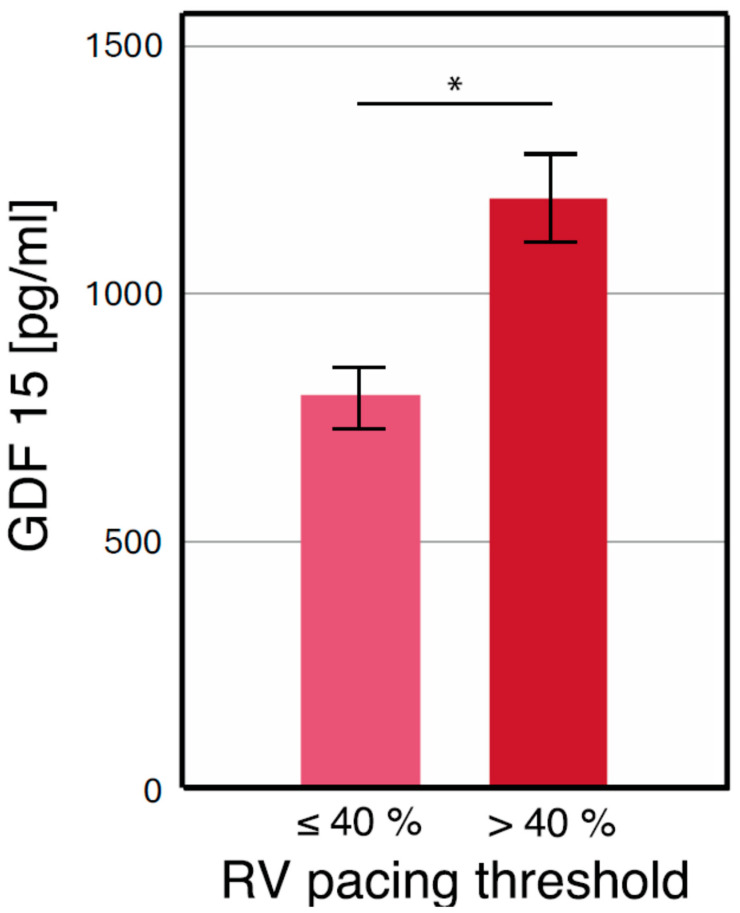
* GDF-15 levels separated by a pacing burden of 40% [26]. This figure has already been presented by our group as a scientific abstract at the national level at the “Jahrestagung der deutschen Gesellschaft für Kardiologie 2023” congress.

**Figure 3:**
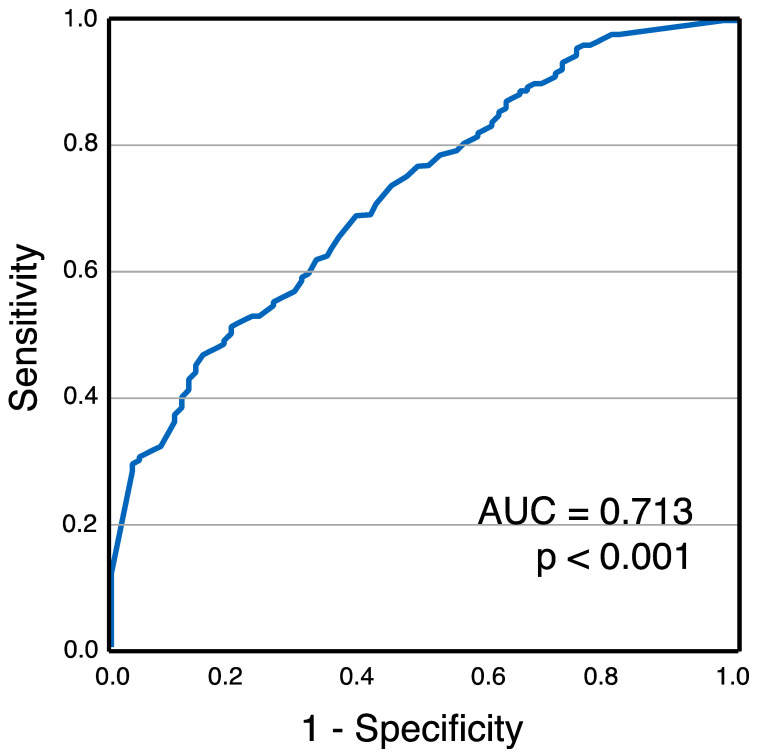
Receiver operating characteristics for GDF-15 levels and chronic RV pacing [26]. This figure has already been presented by our group as a scientific abstract at the national level at the “Jahrestagung der deutschen Gesellschaft für Kardiologie 2023” congress.

Chronic right ventricular (RV) pacing was observed in 67.2% of patients. Baseline patient characteristics are presented in Table 1. When stratified by pacing burden, GDF-15 levels were significantly higher in patients with a burden > 40% compared to those with a burden ≤ 40% (1186 ± 592 pg/mL vs. 789 ± 293 pg/mL, *p* < 0.001) (Figure 2) [26]. Receiver operating characteristic (ROC) analysis identified GDF-15 as a significant marker for chronic RV pacing, with an area under the curve (AUC) of 0.713 (95% confidence interval: 0.650–0.776, *p* < 0.001) (Figure 3) [26].

Patients were further categorized into quartiles based on GDF-15 concentrations (280–672 pg/mL, 672–940 pg/mL, 940–1245 pg/mL, and 1245–3018 pg/mL). A progressive increase in RV pacing burden was observed across quartiles: 45.7 ± 42.8%, 59.2 ± 43.4%, 67.0 ± 40.4%, and 77.8 ± 30.8%, respectively (*p* < 0.001).

A detailed overview of changes from baseline to the 12-month follow-up in GDF-15, NT-proBNP, creatinine-based estimated glomerular filtration rate, and hemoglobin is shown in Table 2.

## 4. Discussion

GDF-15 is a member of the transforming growth factor-beta (TGF-β) superfamily and is involved in various biological processes, including inflammation, metabolism, and cellular stress responses. Its expression increases in response to cellular injury or stress, and it plays key roles in conditions such as cardiovascular disease, cancer, and metabolic disorders [27].

GDF-15 is considered a stress-responsive cytokine that is upregulated in the heart during injury or cardiovascular stress, such as heart failure, myocardial infarction, or pressure overload. It is a biomarker for cardiovascular risk, often elevated in patients with heart diseases, and serves as a prognostic marker for mortality in these patients. GDF-15 has been implicated in fibrosis, where it contributes to tissue remodeling in response to chronic injury. While its exact role in fibrosis is context-dependent, it may have both pro-fibrotic and anti-fibrotic effects depending on the tissue and disease state [23,28,29].

To the best of our knowledge, this is the first study to investigate the role of GDF-15 in chronic RVP. The findings of our study can be summarized as follows:

(1) GDF-15 was significantly increased in patients with increased RVP burden.
(2) GDF-15 could be identified as a potential biomarker for RV pacing.
(3) GDF-15 is an emerging biomarker associated with a variety of cardiovascular pathologies, including heart failure and myocardial stress. The correlation between elevated GDF-15 levels and a right ventricular (RV) pacing >40 suggests that GDF-15 may serve as a valuable marker in the context of PiCM.

### 4.1. Mechanistic Insights

Right ventricular pacing can result in pressure and volume overload in the left ventricle. This is due to inefficient contraction and impaired ejection of blood, further stimulating fibrotic responses. PiCM is a condition where prolonged RV pacing leads to adverse structural and functional cardiac remodeling, typically characterized by dyssynchronous ventricular contraction. This dyssynchrony can increase myocardial stress and contribute to the development of heart failure [30,31].

Given its role as a stress-induced cytokine, GDF-15 levels might be elevated in patients with chronic right ventricular pacing. Elevated GDF-15 levels are indicative of increased cellular stress and inflammatory responses, both of which are critical components in the pathophysiology of PiCM [32]. The association of high GDF-15 with a high RVP burden could potentially be reflective of underlying myocardial stress and the onset of pathological remodeling due to chronic RVP. Therefore, GDF-15 could serve as a biomarker for identifying early cardiac stress in these patients.

### 4.2. Potential Clinical Implications

(1) Early Detection: Utilizing GDF-15 levels as a biomarker could facilitate the early identification of patients at risk for PiCM. Patients presenting with a high RVP burden and elevated GDF-15 levels might be more closely monitored for early signs of ventricular dysfunction, enabling timely intervention.
(2) Risk Stratification: GDF-15 could potentially be integrated into risk stratification models for patients requiring pacemakers. Higher GDF-15 levels in conjunction with elevated pacing burden could identify a subgroup of patients at greater risk for adverse outcomes, thus tailoring follow-up schedules and management strategies accordingly.
(3) Guiding Therapy: For patients with high GDF-15 levels and elevated RVP burden, clinicians might consider alternative pacing strategies, such as CRT or His-bundle pacing, to mitigate the risk of developing PiCM. These alternatives can preserve more natural ventricular contraction patterns, potentially reducing myocardial stress and preventing adverse remodeling.
(4) Monitoring Disease Progression: Serial measurements of GDF-15 could be useful in monitoring the progression of PiCM. Rising GDF-15 levels might indicate worsening myocardial stress and dysfunction, prompting adjustments in pacing strategies or the initiation of heart failure therapies.
(5) Research Implications: Further research is warranted to explore the causal relationships between GDF-15 levels, RVP burden, and the development of PiCM. Longitudinal studies could help establish the predictive value of GDF-15 and elucidate the underlying mechanisms linking pacing-induced myocardial stress with biomarker elevation.

To realize these potential benefits, further research is necessary to validate GDF-15 as a reliable predictor for CRT upgrade candidacy. Prospective studies should focus on the following:

(1) Establishing definitive GDF-15 thresholds that predict adverse outcomes in pacemaker patients.
(2) Evaluating the impact of CRT upgrades in patients identified by elevated GDF-15 levels and high RVP burden.
(3) Investigating the long-term benefits of CRT upgrades guided by GDF-15, including improvements in heart failure symptoms, hospitalization rates, and mortality. The use of GDF-15 as a biomarker to guide CRT upgrades represents another promising future direction in the management of pacemaker patients. This approach could lead to more personalized, timely, and effective interventions, ultimately enhancing patient care and outcomes.

## 5. Limitations

The main limitation of this work is its single-center design and the small sample size. AUC values between 0.7 and 0.8 indicate fair to good discriminative ability. This range indicates a relevant level of discrimination, meaning the model is better than random guessing but may not be reliable for making definitive predictions. In practical applications, this might warrant further investigation or consideration of additional model refinements. Also, other factors such as atrial fibrillation, valvular disease, etc., that cause cardiac stress might have an impact on GDF-15 levels.

## 6. Conclusions

This study suggests that GDF-15 may be a potential biomarker for chronic right ventricular pacing. The association of GDF-15 with a high RV pacing burden is of interest in the context of PiCM. Further studies should investigate the role of GDF-15 as an indicator of myocardial stress and early remodeling in the context of identification, risk stratification, and therapeutic management of PiCM, ultimately improving patient outcomes.

## Figures and Tables

**Table 1:** Baseline patient characteristics.

	Overall n = 265	VP < 40% n = 89	VP ≥ 40% n = 176	*p*-Value
Sex male [%]	60.4	50.6	67.4	0.011
Age [years]	72.1 ± 11.4	68.9 ± 13.7	73.7 ± 9.7	0.005
Body mass index [kg/m^2^]	27.3 ± 4.8	27.9 ± 5.7	26.9 ± 4.4	0.145
Hypertension [%]	81.5	74.2	82.0	0.150
Type II diabetes [%]	21.2	15.7	22.5	0.414
Coronary artery disease [%]	26.4	21.3	29.8	0.188
Chronic kidney disease [%]	23.0	13.5	27.0	0.013
Atrial fibrillation [%]	63.4	46.1	71.9	<0.001
Echocardiographic parameters				
Left ventricular ejection fraction [%]	54.3 ± 5.8	56.6 ± 4.5	53.1 ± 6.2	<0.001
Left ventricular end-diastolic diameter [mm]	46.2 ± 6.1	44.9 ± 5.0	46.9 ± 6.6	0.009
Left atrial volume [mL]	75.8 ± 36.9	62.3 ± 25.1	82.7 ± 40.0	<0.001
Right atrial volume [mL]	49.7 ± 23.8	40.6 ± 10.7	54.2 ± 26.9	<0.001
Pacemaker parameters				
Indication for pacemaker implantation				<0.001
Sick sinus syndrome	20.6	42.7	9.6	
Bradycardia–tachycardia syndrome (incl. brady AF)	16.4	10.0	18.6	
AV-Block II	19.6	13.6	23.1	
AV-Block III	42.8	33.7	47.6	
Other (cardioinhibitory syncope, sinus syndrome)	0.7	0	1.1	
Pacemaker type				<0.001
Single-chamber pacemaker [%]	19.6	5.6	25.8	
Dual-chamber pacemaker [%]	80.4	94.4	74.2	
Pacemaker mode				<0.001
DDD	66.2	71.9	63.3	
VVI	22.6	6.7	30.5	
AAI-DDD	10.2	20.3	5.1	
DDI	1.0	1.0	1.1	
Mode switch [%]	1.0 IQR 1.82	1 IQR 0.09	1 IQR 5.15	0.209
Activation of sensor	38.3	46.1	34.3	0.082
Ventricular pacing [%]	62.5 ± 441.1	8.3 ± 10.3	89.6 ± 16.7	<0.001
Atrial pacing [%]	28.4 ± 34.9	35.6 ± 37.2	24.5 ± 33.2	0.019

IQR = interquartile range.

**Table 2:** Changes in laboratory values upon 12 months’ follow-up.

	Baseline	Follow-Up	*p*-Value
GDF-15 [pg/mL]	988.2 ± 497.5	1324.4 ± 810	0.001
NT-proBNP [pg/mL]	957.6 ± 2303.9	1377.8 ± 3449.5	0.001
eGFR [mL/min]	61.1 ± 11.3	59.4 ± 13.4	0.02
Hemoglobin [g/dL]	13.8 ± 1.6	13.7 ± 1.7	0.102

BNP = brain natriuretic peptide; GFR = glomerular filtration rate.

## Data Availability

The data presented in this study are available on request from the corresponding author.
